# Family resilience and health-related quality of life in children with malignant bone tumors: implications for pediatric oncology nursing

**DOI:** 10.3389/fpsyg.2026.1720767

**Published:** 2026-06-29

**Authors:** Wei Dai, Lan Chen, Zhuqing Wei, Qian Yu, Zhixuan Geng, Ka Yan Ho, Jiawei Pan, Fang Fang

**Affiliations:** 1 Department of Nursing, Shanghai General Hospital, Shanghai Jiao Tong University School of Medicine, Shanghai, China; 2 Shanghai Jiao Tong University School of Nursing, Shanghai, China; 3 The Hong Kong Polytechnic University, Hong Kong, China

**Keywords:** caregiver support, family resilience, health-related quality of life, pediatric bone tumors, pediatric oncology nursing

## Abstract

**Background:**

Pediatric malignant bone tumors impose substantial physical and psychosocial burdens that may compromise health-related quality of life (HRQOL) for both children and caregivers. Family resilience may be protective, yet dyadic evidence in pediatric bone tumor care remains limited.

**Methods:**

This cross-sectional study was conducted at Shanghai General Hospital between May 2023 and July 2024. A total of 134 pediatric patients with malignant bone tumors and their primary caregivers were recruited. Family resilience was assessed using the Chinese version of the Family Resilience Assessment Scale (FRAS). Child health-related quality of life (HRQOL) was assessed using the Pediatric Quality of Life Inventory (PedsQL) 3.0 Cancer Module (child self-report and caregiver proxy-report). Demographic and clinical data were collected from medical records and caregiver reports. Statistical analyses included descriptive statistics, Pearson correlations, and multilevel linear modeling in an Actor–Partner Interdependence Model (APIM) framework, performed using IBM SPSS Statistics version 21.0.

**Results:**

Child HRQOL self-reports were available for 95 children, and caregiver proxy-reports were available for all children (*n* = 134). Family resilience was positively correlated with child self-reported HRQOL (*r* = 0.661, *p* < 0.001) and caregiver proxy-reported child HRQOL (*r* = 0.615, *p* < 0.001), and child self-reported and caregiver proxy-reported HRQOL were highly correlated among paired cases (*r* = 0.899, *p* < 0.001; *n* = 95). In adjusted multilevel models, family resilience predicted higher child HRQOL in both the self-report model (*B* = 0.646, *p* < 0.001; *n* = 95) and the proxy-report model (*B* = 0.627, *p* < 0.001; *n* = 134).

**Conclusion:**

Family resilience was independently associated with HRQOL in pediatric malignant bone tumor dyads. Given the cross-sectional design, causality cannot be inferred; however, family resilience may represent a clinically relevant psychosocial correlate of HRQOL and a potential focus for supportive care. Longitudinal and interventional studies are warranted to clarify directionality and evaluate resilience-oriented supportive approaches.

## Introduction

Malignant bone tumors (e.g., osteosarcoma, Ewing sarcoma, and chondrosarcoma) are rare but clinically challenging pediatric cancers that commonly involve long bones and adjacent soft tissues (Bădilă et al., 2021; Zhou et al., 2025; Steliarova-Foucher et al., 2017). They typically require intensive multimodal treatment and prolonged rehabilitation. Beyond the physical burden, children and their caregivers often experience substantial psychosocial stress, which can compromise HRQOL in both members of the family (Alshehri, 2025; Permana et al., 2025).

In this context, families contend with multiple concurrent stressors, including complex symptom management, prolonged uncertainty, and high-intensity caregiving demands. Caregivers may experience elevated parenting stress and, in some cases, pediatric medical traumatic stress related to the child’s diagnosis and treatment; when persistent, these reactions can undermine caregiver functioning and family communication, with downstream consequences for HRQOL (Çınar et al., 2021; Çınar Özbay et al., 2025). Family resilience refers to a family’s capacity to adapt to adversity, maintain core functioning, and support member growth through shared meaning-making, flexible organization, and open communication (Van Schoors et al., 2015; Yang et al., 2024). Higher resilience has been associated with better psychosocial adaptation and quality of life in pediatric illness contexts, whereas low resilience may contribute to caregiver burnout and family conflict, potentially amplifying vulnerability under chronic stress (Faccio et al., 2018; Suparit et al., 2023). Notably, malignant bone tumors often entail distinctive challenges—such as major surgical decisions (limb salvage versus amputation), functional impairment, and perceived appearance concerns—which can intensify shared stress and place greater demands on coordinated family coping.

Existing pediatric oncology studies suggest that family resilience or family functioning is related to psychosocial adjustment and quality-of-life outcomes (Van Schoors et al., 2015; Faccio et al., 2018); however, Most existing evidence comes from mixed cancer populations and typically examines children and caregivers separately, with dyadic analytical approaches remaining scarce. Moreover, given the function- and appearance-related sequelae and key surgical decisions (e.g., limb salvage vs. amputation) unique to malignant bone tumors, family coping processes may differ from those in other pediatric cancers; whether family resilience shows an independent association with HRQOL in this context—beyond socioeconomic and clinical factors—remains insufficiently examined.

Despite growing attention to resilience in pediatric oncology, dyadic resilience-focused evidence in malignant bone tumor care is limited. Because children and caregivers experience the illness together and their responses mutually shape each other, examining family resilience and HRQOL from a dyadic perspective is warranted. Therefore, this study aimed to investigate the association between family resilience and child HRQOL in child–caregiver dyads with malignant bone tumors, and to test whether family resilience predicts child HRQOL independently of socioeconomic and clinical factors. We hypothesized that: (1) higher family resilience would be associated with better child HRQOL based on both child self-reports and caregiver proxy-reports; and (2) family resilience would predict child HRQOL independently of socioeconomic and clinical factors.

## Materials and methods

### Participants and procedure

**Figure 1:**
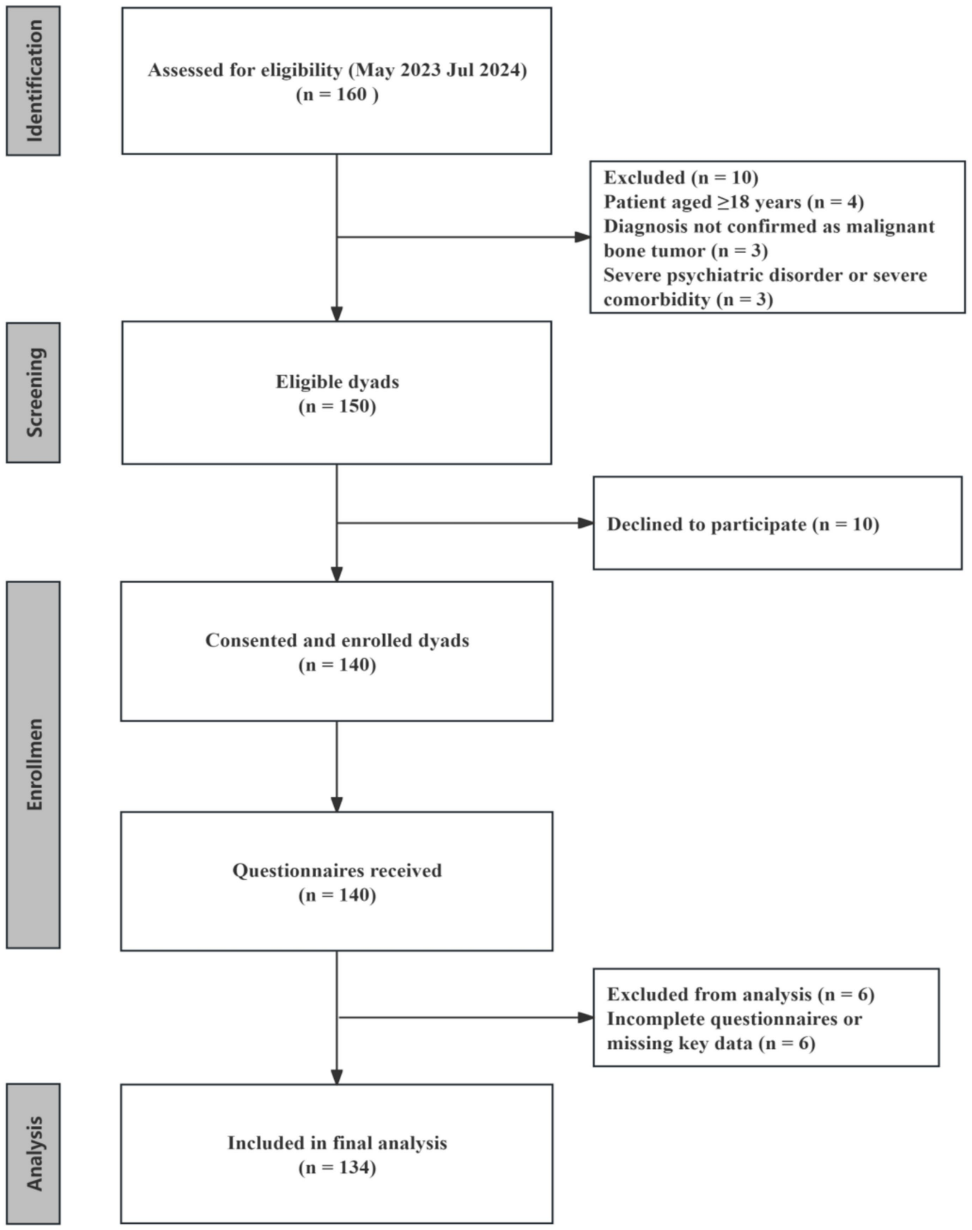
Flow diagram of participant recruitment and selection. Counts refer to child–caregiver dyads (one pediatric patient paired with one primary caregiver).

This single-center cross-sectional study was conducted between May 2023 and July 2024 at a regional bone tumor center (Shanghai General Hospital), Shanghai, China. Using convenience sampling, we approached 160 child–caregiver dyads during the study period; 150 met the inclusion criteria. Of these, 10 declined to participate. Questionnaires were received from 140 dyads; 6 dyads were excluded from analysis due to incomplete questionnaires or missing key data, resulting in 134 dyads included in the final analysis (overall response rate: 83.8%; see Figure 1). The study was approved by the Ethics Committee of Shanghai General Hospital (2022SQ508).

### Inclusion criteria

- Inclusion criteria for pediatric patients were: (1) < 18 years; (2) confirmed diagnosis of malignant bone tumor; (3) enrolled at varying time points after initial diagnosis, and the interval from initial diagnosis to enrollment (time since initial diagnosis, months) was recorded; and (4) no psychiatric disorders or other serious comorbidities.
- Inclusion criteria for caregivers were: (1) ≥ 18 years; (2) primary caregivers of pediatric patients; (3) no psychiatric disorders or other serious comorbidities; and (4) willingness to participate.

### Exclusion criteria

- Patients with mental disorders or severe complications.
- Families experiencing other major traumatic events.
- Caregivers who were professional attendants.

Sample size was estimated *a priori* using G*Power 3.1. Based on a medium effect size (*f*^2^ = 0.15), *α* = 0.05, power = 0.80, and 8 predictors, a minimum of 114 dyads was required. Our sample of 134 dyads exceeds this threshold.

Data were collected via self-administered questionnaires completed separately by children and caregivers, with standardized instructions provided by three trained research assistants to minimize investigator bias. Dyads were included if caregiver questionnaires were complete. For HRQOL, child self-report and caregiver proxy-report were treated as reporter-specific outcomes rather than pooled into a single score. When a child could not complete a valid self-report, the dyad was retained for proxy-report analyses and the child self-report was coded as missing. Written parental consent and child assent were obtained. Clinical characteristics were extracted from hospital medical records.

### Measurement

#### Sociodemographic and clinical characteristics

Demographic variables included gender, age, employment status, marital status, educational level, household monthly income, and religious beliefs. Clinical variables collected via chart review included tumor type, location, treatment, time since diagnosis, initial symptoms, medical expenses, and payment method.

For HRQOL assessment, we used the Pediatric Quality of Life Inventory (PedsQL) 3.0 Cancer Module child self-report and caregiver proxy-report forms. Child self-report was administered to children aged 8–16 years who were able to complete the questionnaire independently. Caregivers completed the proxy-report form for all children and were instructed not to assist or influence the child’s answers. For children aged 3–7 years (*n* = 30) and for older children who were unable to provide a valid self-report due to pain, fatigue, or weakness (*n* = 9), the caregiver proxy-report was used as the child HRQOL assessment. Overall, valid child self-reports were obtained from 95 children (70.9%), and caregiver proxy-reports were completed for all children (*n* = 134); therefore, 39 children (29.1%) had proxy-report only. Self-reports and proxy-reports were analyzed as parallel reporter-specific outcomes and were not pooled into a single HRQOL measure.

#### Family resilience

Family resilience was assessed using the 32-item Chinese version of the Family Resilience Assessment Scale (FRAS-C) (Dong et al., 2021), which includes three subscales: family communication and problem solving (FCPS), utilization of social resources (USR), and maintaining a positive outlook (MPO). Items were rated on a 4-point Likert scale (1 = strongly disagree to 4 = strongly agree), with total scores ranging from 32 to 128. Higher scores indicate greater family resilience. Cronbach’s *α* was 0.846 in this study.

#### Perceived social support

Caregivers’ perceived social support was measured using the Chinese 12-item Perceived Social Support Scale (PSSS) (Zimet et al., 1990) comprising internal family (4 items) and external family (8 items) factors. Items were rated on a 7-point Likert scale (1 = very strongly disagree to 7 = very strongly agree), with total scores ranging from 12 to 84. Higher scores indicate greater perceived support. Cronbach’s *α* was 0.924.

#### Psychological resilience

Caregivers’ psychological resilience was measured using the 10-item Connor–Davidson Resilience Scale (CD-RISC-10) (Ye et al., 2017), scored 0 (“not true at all”) to 4 (“true nearly all the time”), with total scores ranging from 0 to 40. Higher scores indicate better individual resilience. Cronbach’s *α* was 0.913.

#### Caregiver burden

Caregiver burden was assessed using the 22-item Zarit Burden Interview (ZBI) (Adib-Hajbaghery and Ahmadi, 2019), including personal and responsibility burden. Items were scored 0 (almost none) to 4 (always), with total scores ranging from 0 to 88; higher scores indicate greater burden. Cronbach’s α was 0.901.

#### Pediatric Quality of Life Inventory 3.0 Cancer Module

The PedsQL 3.0 Cancer Module assesses cancer-specific HRQOL in children and young adults (2–25 years) using age-appropriate child self-report and parent proxy-report forms. It includes 27 items across eight domains: pain and hurt, nausea, procedural anxiety, treatment anxiety, worry, cognitive problems, perceived physical appearance, and communication. Items are rated on a 5-point scale (or a 3-point scale with visual aids for ages 5–7), reverse-scored, and linearly transformed to a 0–100 scale, with higher scores indicating better HRQOL. We selected this module because it captures symptom- and treatment-related domains that are particularly relevant to pediatric malignant bone tumor care (e.g., pain, nausea, treatment-related anxiety, and perceived physical appearance).

### Statistical analysis

Continuous variables were grand-mean centered. Dyad-level ICCs were calculated, and multilevel models with random intercepts were used to account for dyadic interdependence. Child HRQOL based on caregiver proxy-report was analyzed in the full sample (*n* = 134), while child self-report HRQOL was analyzed only in children with valid self-reports (*n* = 95). Self-reports and proxy-reports were treated as parallel reporter-specific outcomes rather than pooled.

Agreement between child self-report and caregiver proxy-report was assessed using Pearson correlation among dyads with both reports available. Agreement between child self-report and caregiver proxy-report was assessed using Pearson correlation among the 95 dyads with both reports available; children with proxy-report only (*n* = 39) were retained in proxy-report analyses but excluded from the agreement analysis. Descriptive statistics summarized participant characteristics. Bivariate associations among continuous variables were examined using Pearson correlations. Univariable HRQOL comparisons used independent-samples t-tests for dichotomous predictors and one-way ANOVA for categorical predictors with three or more groups. Associations between categorical variables were examined using chi-square or Fisher’s exact tests. To account for dyadic interdependence, multilevel linear models were fitted within the Actor–Partner Interdependence Model framework with random intercepts for dyads. Two parallel outcome models were estimated: child self-report HRQOL (*n* = 95) and caregiver proxy-report HRQOL (*n* = 134). Covariates were prespecified based on clinical relevance and prior literature. Core psychosocial predictors included family resilience (FRAS), perceived social support (PSSS), caregiver resilience (CD-RISC-10), and caregiver burden (ZBI). Model assumptions were checked using residual plots; multicollinearity was assessed with variance inflation factors (all VIFs <2). Results are reported as unstandardized regression coefficients (B) with standard errors, 95% confidence intervals, and exact *p*-values (*p* < 0.001 reported as such). Missing data were handled using listwise deletion; item-level missingness was <5% across scales, and Little’s MCAR test was non-significant (*p* > 0.05). Statistical analyses were performed using IBM SPSS Version 21.0, with significance set at *p* < 0.05.

## Results

### Demographic and clinical characteristics

Table 1 summarizes patient and caregiver characteristics. Median ages were 12 years (patients) and 39 years (caregivers). Most caregivers were mothers (110/134, 82.1%), whereas fathers accounted for 24/134 (17.9%). Because this imbalance was substantial, separate analyses of maternal and paternal resilience were not performed. Osteosarcoma was the most common tumor type (85.8%), primarily located in the limbs (79.9%). Limb-salvage surgery was performed in 91% of patients.

**Table 1:** Sample characteristics (*N* = 134).

Variables	Child/*n* (%)	Caregiver/*n* (%)
Sex		
Male	82 (61.2)	24 (17.9)
Female	52 (38.8)	110 (82.1)
Age (year)		
M ± SD	12.39 ± 3.48	40.32 ± 6.49
Median	12	39
Types of cancer		
Osteosarcoma	115 (85.8)	–
Other	19 (14.2)	–
Location of cancer		
Limbs	107 (79.9)	–
Other	27 (20.1)	–
Surgery		
Segment resection	65 (48.5)	–
Segment resection+ Joint replacement	69 (51.5)	–
Amputation or not		
Amputation	12 (9.0)	–
Primary surgery or not		
Primary surgery	85 (63.4)	–
Initial symptom		
Pain	90 (67.2)	–
Other	44 (32.8)	–
Number of children		
1	52 (38.8)	–
2 and more	82 (61.2)	–
Time since first diagnosis		
≤20^+^	67 (50.0)	–
>20	67 (50.0)	–
Marital status		
Married	–	126 (94.0)
Divorce and other	–	8 (6.0)
Monthly household income		
≤3,000	–	59 (44.0)
>3,000	–	75 (56.)
Education		
≤Middle school	–	66 (49.2)
>Middle school	–	68 (50.8)
Employment status		
Unemployed	–	59 (44.0)
Other	–	75 (56.0)
Payment		
NCMS	–	73 (54.5)
URBMI	–-	56 (42.8)
At own expenses	–	5 (2.7)
Religion		
Buddhism	–	13 (9.7)
Christian	–	7 (5.2)
No	–	114 (85.1)

M, Mean; SD, Standard deviation; NCMS, New rural cooperative medical system; URBMI, Urban residents’ basic medical insurance system. +: The median time since first diagnosis was 20 months.

### HRQOL assessment and domain scores

Child HRQOL was assessed using the PedsQL 3.0 Cancer Module. Of the 134 children, 95 (70.9%) completed the child self-report form. Caregiver proxy-reports were available for all children; therefore, 39 children (29.1%) had proxy-report only (children aged 3–7 years, *n* = 30; and children aged ≥8 years who were unable to complete a valid self-report due to pain, fatigue, or weakness, *n* = 9). Domain-specific scores of the PedsQL 3.0 Cancer Module are provided in Table 2.

**Table 2:** PedsQL 3.0 Cancer Module domain scores (mean ± SD).

Domain	Child self-report (*n* = 95) (Mean ± SD)	Caregiver proxy-report (*n* = 134) (Mean ± SD)
Pain and hurt	54.34 ± 22.35	56.75 ± 21.16
Nausea	48.94 ± 21.84	49.91 ± 23.59
Procedural anxiety	48.51 ± 25.03	49.25 ± 24.41
Treatment anxiety	56.09 ± 23.97	53.30 ± 24.95
Worry	49.75 ± 24.46	40.86 ± 26.23
Cognitive problems	64.97 ± 21.34	62.24 ± 21.84
Perceived physical appearance	51.12 ± 27.93	51.74 ± 25.20
Communication	66.48 ± 21.32	65.98 ± 21.17
Total score (mean of 27 items)	79.63 ± 31.80	82.41 ± 28.43

Scores were transformed to a 0–100 scale; higher scores indicate better HRQOL. Total score is the mean of the 27 items (0–100 transformed scale).

### Bivariate analysis between HRQOL and family resilience variables

At the individual level, child HRQOL (self-report; *n* = 95) was positively correlated with FRAS (*r* = 0.661, *p* < 0.001), PSSS (*r* = 0.362, *p* < 0.001), and CD-RISC-10 (*r* = 0.298, *p* = 0.003), and negatively correlated with caregiver burden (*r* = −0.584, *p* < 0.001). Caregiver proxy-reported child HRQOL (*n* = 134) correlated positively with FRAS (*r* = 0.615, *p* < 0.001), PSSS (*r* = 0.313, *p* < 0.001), and CD-RISC-10 (*r* = 0.279, *p* = 0.001), and negatively correlated with caregiver burden (*r* = −0.554, *p* < 0.001). Among dyads with both child self- and caregiver proxy-reports (*n* = 95), child self-reported and caregiver proxy-reported HRQOL were highly correlated (*r* = 0.899, *p* < 0.001; see Table 3), suggesting substantial convergence between reporters in paired cases, although the two reports were analyzed separately rather than treated as interchangeable.

**Table 3:** Pearson correlations among study variables (*N* = 134).

Variables	HRQOL		FRAS	PSSS	Resilience (CD-RISC-10)	Caregiver burden (ZBI)
	Child HRQOL (self-report)	Child HRQOL (caregiver proxy-report)				
Child HRQOL (self-report)	1	0.899 (*p* < 0.001)	0.661 (*p* < 0.001)	0.362 (*p* < 0.001)	0.298 (*p* = 0.003)	−0.584 (*p* < 0.001)
Child HRQOL (caregiver proxy-report)		1	0.615 (*p* < 0.001)	0.313 (*p* < 0.001)	0.279 (*p* = 0.001)	−0.554 (*p* < 0.001)
FRAS			1	0.540 (*p* < 0.001)	0.459 (*p* < 0.001)	−0.640 (*p* < 0.001)
PSSS				1	0.456 (*p* < 0.001)	−0.497 (*p* < 0.001)
Resilience (CD-RISC-10)					1	−0.307 (*p* < 0.001)
Caregiver burden (ZBI)						1

HRQOL, health-related quality of life; FRAS, Family Resilience Assessment Scale; PSSS, Perceived Social Support Scale; CD-RISC-10, 10-item Connor–Davidson Resilience Scale; ZBI, Zarit Burden Interview. Correlations involving child HRQOL (self-report) were based on available child self-reports (*n* = 95); all other correlations were based on the full sample (*n* = 134). Exact *p*-values are shown; values < 0.001 are reported as *p* < 0.001.

### Univariate analyses of clinical factors associated with HRQOL

Children aged ≤12 years reported higher child self-reported HRQOL (*p* = 0.022). Children without amputation reported higher child self-reported HRQOL (*p* = 0.034). Higher monthly household income (>3,000 CNY) and younger caregiver age (≤39 years) were associated with higher child HRQOL in both self-reports (*n* = 95) and proxy-reports (*n* = 134) (all *p* < 0.05). In addition, higher caregiver education (> middle school) was associated with higher child self-reported HRQOL (*p* = 0.024; see Table 4).

**Table 4:** Univariate analyses of factors associated with HRQOL in child–caregiver dyads (*N* = 134).

Variables		HRQOL (M ± SD)				Variables		HRQOL (M ± SD)			
		Child HRQOL (self-report)	*p*	Child HRQOL (caregiver proxy-report)	*p*			Child HRQOL (self-report)	*p*	Child HRQOL (caregiver proxy-report)	*p*
Gender	Male	54.55 ± 15.58	0.478	52.46 ± 16.13	0.180	Gender	Male	55.27 ± 14.04	0.948	54.61 ± 13.38	0.770
	Female	56.58 ± 17.00		56.39 ± 16.91			Female	55.04 ± 16.33		53.54 ± 16.90	
Age	≤12^**+**^	58.20 ± 17.31	0.022	57.88 ± 17.52	0.002	Age	≤39+	58.14 ± 16.33	0.038	56.86 ± 15.96	0.037
	>12	51.81 ± 13.85		49.19 ± 13.81			>39	52.37 ± 15.46		50.94 ± 16.61	
Types of cancer	Osteosarcoma	55.32 ± 15.94	0.983	54.12 ± 15.81	0.820	Marital status	Married	55.93 ± 16.16	0.093	54.19 ± 16.80	0.576
	Other	55.41 ± 17.58		53.19 ± 20.62			Divorce and other	46.06 ± 12.85		50.81 ± 10.47	
Location of cancer	Limbs	56.26 ± 15.91	0.188	54.80 ± 16.61	0.258	Monthly household income	≤3,000	51.88 ± 16.70	0.027	50.79 ± 16.73	0.046
	Other	51.68 ± 16.71		50.77 ± 15.88			>3,000	58.06 ± 15.20		56.50 ± 15.95	
Surgery	Segment resection	54.41 ± 17.08	0.520	52.03 ± 17.14	0.184	Education	≤Middle school	52.16 ± 15.70	0.024	51.53 ± 15.33	0.090
	Other	56.21 ± 15.22		55.83 ± 15.75			>Middle school	58.42 ± 16.02		56.37 ± 17.32	
Amputation	Yes	45.96 ± 18.10	0.034	47.89 ± 15.08	0.180	Employment status	Employed	57.04 ± 15.68	0.279	55.64 ± 16.61	0.304
	No	56.26 ± 15.69		54.59 ± 16.55			Unemployed	54.00 ± 16.43		52.68 ± 16.38	
Primary surgery	Yes	57.40 ± 15.70	0.050	55.81 ± 16.13	0.092	Payment	NCMS	56.33 ± 16.74	0.360	55.78 ± 16.40	0.715
	No	51.75 ± 16.36		50.82 ± 16.79			URBMI	53.99 ± 15.83		52.08 ± 17.08	
Initial symptom	Pain	55.52 ± 15.58	0.852	53.41 ± 16.09	0.564		At own expenses	55.93 ± 9.94		49.07 ± 6.45	
	Other	54.96 ± 17.34		55.17 ± 17.40		Religion	Buddhism	63.18 ± 17.94	0.900	62.78 ± 19.31	0.429
Number of children	1	53.68 ± 14.61	0.345	51.69 ± 16.23	0.201		Christian	59.00 ± 16.09		55.42 ± 12.61	
	2 and more	56.39 ± 17.01		55.44 ± 16.58			No	54.22 ± 15.77		52.90 ± 16.18	
Time since first diagnosis	≤20+	57.01 ± 14.98	0.232	55.62 ± 16.70	0.254						
	>20	53.67 ± 17.13		52.36 ± 16.24							

Child HRQOL self-report analyses were based on available child self-reports (*n* = 95); proxy-report analyses were based on the full sample (*n* = 134). “+” indicates the median cut-off used for grouping (children: 12 years; caregivers: 39 years; time since diagnosis: 20 months). *p* values were obtained using independent-samples *t* tests (two-sided) for two-group comparisons and one-way ANOVA for variables with ≥3 categories; *p* values are reported once per variable (overall comparison). M, mean; SD, standard deviation; NCMS, New Rural Cooperative Medical Scheme; URBMI, Urban Residents’ Basic Medical Insurance.

### Multilevel models analyses predicting HRQOL

Multilevel modeling identified both patient- and caregiver-level factors influencing child HRQOL (Table 5). Family resilience (FRAS) was positively associated with child HRQOL in both the self-report model (*B* = 0.646, 95% CI 0.406–0.886, *p* < 0.001) and the proxy-report model (*B* = 0.627, 95% CI 0.380–0.874, *p* < 0.001). Caregiver burden (ZBI) was negatively associated with child HRQOL in both the self-report model (*B* = −0.247, 95% CI − 0.419 to −0.075, *p* = 0.005) and the proxy-report model (*B* = −0.279, 95% CI − 0.457 to −0.101, *p* = 0.003).

**Table 5:** Adjusted multilevel model predicting child HRQOL (self-report; *n* = 95) and child HRQOL (caregiver proxy-report; *n* = 134) (Family resilience).

Variables	Child HRQOL (self-report; *n* = 95)				Child HRQOL (caregiver proxy-report; *n* = 134)			
	*B*	SE	95% *CI*	*p*	*B*	SE	95% *CI*	*p*
Age	−2.614	2.139	(−6.893, 1.665)	0.224	−1.928	2.285	(−6.407, 2.551)	0.400
No amputation (vs. amputation)	4.392	3.685	(−2.979, 11.763)	0.236				
Monthly household income >3,000 RMB (vs ≤ 3,000)	−1.551	2.555	(−6.662, 3.560)	0.545	−1.572	2.385	(−6.247, 3.103)	0.511
Education > Middle school (vs ≤ Middle school)	−0.474	2.681	(−5.837, 4.889)	0.860	—	—	—	—
Family resilience								
FRAS	0.646	0.120	(0.406, 0.886)	**<0.001**	0.627	0.126	(0.380, 0.874)	**<0.001**
PSSS	−0.046	0.097	(−0.240, 0.148)	0.634	−0.112	0.101	(−0.310, 0.086)	0.272
Resilience (CD-RISC-10)	0.050	0.180	(−0.310, 0.410)	0.780	0.042	0.192	(−0.334, 0.418)	0.826
Caregiver burden (ZBI)	−0.247	0.086	(−0.419, −0.075)	**0.005**	−0.279	0.091	(−0.457, −0.101)	**0.003**

B, unstandardized regression coefficient; SE, standard error; CI, confidence interval. FRAS, Family Resilience Assessment Scale; PSSS, Perceived Social Support Scale; CD-RISC-10, 10-item Connor–Davidson Resilience Scale; ZBI, Zarit Burden Interview. Coefficients are adjusted for the covariates shown in the table. For dichotomous predictors, effects are reported for the category listed versus the reference category (shown in parentheses). Self = child self-report; Proxy = caregiver proxy-report. Bold values indicate statistically significant regression coefficients (*p* < 0.05).

## Discussion

### Family resilience as a determinant of health-related quality of life

In this study, family resilience was significantly associated with child HRQOL (child self-report: *B* = 0.646; caregiver proxy-report: *B* = 0.627) (Li et al., 2022). Symptom/treatment domains scored lower than communication domains, indicating pain, treatment burden, and appearance concerns remain central challenges. Caregiver distress includes parenting stress and trauma-related responses, affecting family interactions and child well-being (Çınar et al., 2021; Çınar Özbay et al., 2025). Family resilience helps maintain coordinated routines, shared problem-solving, and supportive communication. This aligns with the VSA model (McNulty et al., 2021), and resilience may function as a stabilizing family resource in pediatric oncology (Silva et al., 2021). Children with malignant bone tumors face pain, functional impairment, and prognostic uncertainty (Park et al., 2021); more resilient families better adjust caregiving roles and maintain positive outlook. Findings are consistent with prior chronic illness research (Baker and Claridge, 2022), remaining significant after adjusting for socioeconomic/clinical factors, though residual confounding (e.g., caregiver mental health, treatment intensity) cannot be excluded. The results also support resilience frameworks emphasizing shared beliefs, flexibility, and mutual support (Saltzman et al., 2011). Family resilience is a dynamic process shaping responses to illness-related stress. Interventions strengthening meaning-making, communication, and collaborative coping may be relevant (Simard et al., 2024). Clinical assessment of family resilience complements symptom/physical rehabilitation approaches (Mooney-Doyle et al., 2020), applicable across care stages (Ayoub et al., 2024), aligning with family-centered nursing in pediatric oncology (Mcharo et al., 2023). Longitudinal and interventional studies are still needed to clarify causality.

### Interacting pathways between family functioning and psychosocial well-being

The association between family resilience and HRQOL may involve multiple psychosocial pathways. Consistent with prior work, family cohesion and communication are linked to better psychosocial adjustment in families of children with cancer (Long and Marsland, 2011), which supports our finding that higher resilience was associated with better HRQOL. Positive meaning-making and open communication have also been identified as key mechanisms (Park et al., 2022), and resilience-promoting programs show potential value (Park et al., 2022). Our data further suggest a pathway through caregiver strain: higher resilience was associated with lower caregiver burden, which in turn was linked to higher child HRQOL, supporting the view that shared beliefs and organized functioning transform distress into collaborative coping (Walsh, 2016). This aligns with evidence that parental coping is closely tied to children’s emotional adjustment during treatment (Compas et al., 2015). The strong correlation between child self-reports and caregiver proxy-reports indicates convergence, though proxy reports are not equivalent to direct child reports (Lyons and Lee, 2020). Prior research has also linked family resilience to lower psychological symptom burden and better recovery in critical illness (Sottile et al., 2016), as well as sustained well-being in pediatric chronic illness (Herbell et al., 2020). Our study did not measure physiological indicators but supports the interpretation that resilience contributes to daily adaptation and perceived well-being. Finally, sociocultural norms in our Chinese sample may influence caregiving roles and responses to adversity, though these factors were not directly measured and require future investigation in more diverse, multicenter samples.

### Integrating family resilience into pediatric oncology nursing practice

The present findings suggest that family resilience has practical relevance for pediatric oncology nursing. Because resilience was independently associated with child HRQOL in both child and proxy reports, it may be useful to assess resilience alongside symptom management and rehabilitation. Resilience-oriented interventions can improve well-being and communication in chronically stressed families (Saltzman et al., 2016), and resilience-focused family education is associated with better parental coping and child emotional stability (Herbell et al., 2020). Although not yet established in pediatric bone tumor populations, these findings support resilience-informed supportive care. Nursing support may include guided communication, coping support, psychoeducation, and attention to caregiver strain. Psychosocial risk screening at diagnosis can help identify distressed families and facilitate timely referral (Kazak et al., 2011), with nurses playing a coordinating role across the illness trajectory. These implications should be interpreted cautiously. Family-centered pediatric oncology nursing already emphasizes child and family support (Mooney-Doyle et al., 2020); the present findings highlight resilience as a clinically relevant process, applicable across key stages including diagnosis, perioperative recovery, rehabilitation, and repeated treatment cycles (Ayoub et al., 2024). However, due to the cross-sectional design, implications remain hypothesis-generating, and longitudinal and interventional studies are needed before firm practice recommendations can be made (Mcharo et al., 2023).

### Measurement considerations

Although the instruments are widely used, several validity issues remain. Cronbach’s alpha reflects internal consistency but not full construct validity in this population. Family resilience was reported only by the primary caregiver (mostly mothers), not independently by both parents.

Child self-reports and caregiver proxy-reports may differ systematically; we analyzed them separately rather than pooling, and examined agreement only in the 95 dyads with both reports. The strong correlation in that subgroup suggests concordance but does not formally validate proxy reports.

Additionally, 29.1% of children had proxy-report only, and most caregivers were mothers, limiting examination of reporter bias or maternal versus paternal perspectives. Future studies should assess cross-informant agreement more comprehensively with balanced samples.

## Conclusion and limitations

This study found that higher family resilience was independently associated with better child HRQOL in malignant bone tumor dyads, based on both child self-reports and caregiver proxy-reports. Family resilience may be a clinically relevant psychosocial correlate of adaptation in pediatric oncology families and could inform supportive care planning, though the findings are associative rather than causal. Several limitations should be noted. The cross-sectional, single-center convenience sample limits causal inference and generalizability, and residual confounding including caregiver mental health, access to psychosocial services, and treatment phase or intensity cannot be excluded. Self-report and proxy-report measures may introduce rater bias and shared method variance. Additionally, most caregivers were mothers, so separate analyses of maternal and paternal perspectives were not feasible. Due to the highly unbalanced gender distribution (mothers 82.1%, fathers 17.9%), we were unable to conduct separate analyses or comparisons of family resilience, psychological resilience, and caregiver burden between mothers and fathers. Gender differences in resilience and caregiving patterns could not be explored in this study. Future studies with a more balanced sample of maternal and paternal caregivers are warranted to examine gender-specific resilience characteristics. Caregiver proxy-reports may be subject to under- or overestimation, though they showed high correlation with child self-reports. Future research should prioritize dyadic longitudinal designs to clarify temporal directionality and mechanisms, as well as intervention studies to examine whether resilience-focused family support can improve HRQOL across key treatment stages.

### Clinical implications

Clinicians may consider assessing family resilience as part of psychosocial evaluation and using resilience-informed family support to complement symptom management and rehabilitation. Because the present study was cross-sectional, these implications should be regarded as hypothesis-generating and require confirmation in longitudinal and interventional studies.

## Data Availability

The raw data supporting the conclusions of this article will be made available by the authors, without undue reservation.
